# InSilico DB: an online platform to collaboratively structure and export publicly available datasets from the Gene Expression Omnibus database

**DOI:** 10.1186/gb-2011-12-s1-p33

**Published:** 2011-09-19

**Authors:** A Coletta, C Molter, R Duqué, D Steenhoff, J Taminau, V de Schaetzen, C Lazar, S Meganck, A Nowé, H Bersini, D Weiss

**Affiliations:** 1 IRIDIA, Université libre de Bruxelles, Brussels 1050, Belgium; 2 COMO, Vrije Universiteit Brussel, Brussels 1050, Belgium

## 

There are more than 20,000 genomic studies comprising 500,000 samples freely available in the Gene Expression Omnibus (GEO) database [1]. However, accessing these data requires complex computational steps, including structuring and formatting the clinical vocabulary used to annotate the samples. These complex steps hinder the accessibility of genomic datasets through visualization and analysis software platforms, such as GenePattern and R/Bioconductor, therefore hampering the pace of research.

InSilico DB [2] is an online platform that provides a complete collaborative solution for structuring and formatting clinical annotations from GEO, making GenePattern and R datasets one click away for researchers.

InSilico DB has made available powerful and intuitive online curation tools to structure the metadata of GEO datasets. The database is automatically updated daily, through GEO import pipelines. Datasets can have multiple annotations given by different users, and one user can have multiple versions of an annotation to suit different experimental questions.

The InSilico DB platform supports datasets from Affymetrix human gene expression platforms, which account for 2,900 studies comprising 110,000 samples, making InSilico DB the largest public database of manually curated human gene expression samples. In addition to the web interface, InSilico DB offers programmatic access through an R/Bioconductor package [3].

Future releases of InSilico DB will include Illumina RNA-Seq platform data and Affymetrix mouse gene expression data.
